# The role of masks and respirator protection against SARS-CoV-2

**DOI:** 10.1017/ice.2020.83

**Published:** 2020-03-20

**Authors:** Qiang Wang, Chaoran Yu

**Affiliations:** 1 Fudan University Shanghai Cancer Center, Fudan University, Shanghai, 200025, PR China; 2 Department of Oncology, Shanghai Medical College, Fudan University, Shanghai, 200025, PR China

*To the Editor*— The outbreak of COVID-19, the novel coronavirus SARS-CoV-2 infection, was first reported on December 31, 2019, in Wuhan, a central city in China. The SARS-CoV-2 virus has infected >30,000 people in a very short time, with hundreds of deaths.^1^ COVID-19 continues to be a flaming infectious disease across the world. However, many details of the biological features of this virus remain largely unknown.

SARS-CoV-2 is the third coronavirus to have threatened global public health in the past 20 years, following severe acute respiratory syndrome coronavirus (SARS-CoV) in 2002 and Middle East respiratory syndrome coronavirus (MERS-CoV) in 2012.^2^ In an updated COVID-19 report, Wang et al^3^ indicated that the median age of death was 75 years, and fever (64.7% of deaths) and cough (52.9% of deaths) were identified as initial clinical manifestation.^3^ Genomic characterization of samples from 9 COVID-19 patients indicated that SARS-CoV-2 had 88% identity with 2 bat-derived SARS-like coronaviruses (bat-SL-CoVZC45 and bat-SL-CoVXC21), whereas these bat viruses had 79% identity with SARS-CoV and 50% identity with MERS-CoV.^4^ A phylogenetic analysis has indicated that SARS-CoV-2 belongs to the genus *Betacoronavirus* and the subgenus *sarbecovirus*.^4^

Given that large-scale spread of this virus is now occurring around the world, the identification of cases and the containment of possible routes of spread have become a priority. Increasing risk has narrowed the window of opportunity for effective abatement of COVID-19. Containing an outbreak becomes much more complicated and challenging when hospitalized populations are exposed. Notably, Zhou et al^5^ recommended urgent interventions for the protection of Chinese healthcare workers against SARS-CoV-2. In fact, this warning raised attention regarding the role of daily-use N95 respirators and masks during this pandemic.

Given the similarity of SARS-CoV-2 and SARS-CoV, initial political recommendations in China highlighted the use of masks and N95 respirators for protection against SARS-CoV-2. Wearing masks and respirators and self-isolation at home has been issued as a practice guideline for public in China. Of 213 medical staff with no mask, 10 were infected by SARS-CoV-2, but none of the 278 medical staff wearing N95 respirators was infected.^6^ Interestingly, a higher risk of infection has been noted in male professionals, and the study by Wang et al^6^ highlights the essential role of occupational protection.

However, evidence-based guidelines remain scarce. By definition, N95 respirators are designed to reduce oral entry of small airborne particles with clear filtration requirements. The respirator must fit tightly to wearerʼs face with limited seal leakage to be effective. Medical masks, also known as surgical masks, are used to protect the wearers from microorganism transmission, specifically during hand-to-face contact and large droplets and sprays. In fact, the comparably loose wearing of medical masks does not efficiently prevent the entry of small airborne particles. Both masks and N95 respirators are used to protect against airborne viral pathogens such as SARS-CoV and influenza virus. Therefore, it has been reasonable to initiate widespread mask and respirator protocols across China when facing a coronavirus similar to SARS-CoV.

However, the question remains: Does N95 respirator demonstrate better outcomes than medical masks? In fact, the clinical effectiveness of N95 respirators compared with medical masks in protecting against respiratory infection transmission has not been fully assessed. Particularly, quantified protection analyses among healthcare individuals close to patients suspected of respiratory illness are limited.^7^ In a randomized clinical trial of 2,862 healthcare personnel that analyzed the effectiveness of N95 respirators and medical masks, Radonovich et al^8^ reported no significant difference in the incidence of laboratory-confirmed influenza between these 2 types of equipment. Notably, disease-bound features remain another uncertainty limiting the direct translational protective implementation. Experience with the influenza A virus may offer an explanation.^2^ The altered specificity of influenza virus-targeting receptor has resulted in a changing disease burden due to a shift from the lower to the upper respiratory tract. The H1N1 virus targets the upper respiratory tract, with endemic populations and comparably mild disease, whereas H7N9 targets the lower respiratory tract, with fewer cases of human-to-human transmission.^2^ However, without sufficient mechanistic evidence, whether this lesson can be translated into COVID-19 remains unknown.

Nonetheless, the long-term protective role of both masks and respirators is another emerging concern given the worldwide spread of SARS-CoV-2. A previous review of published literature, including 67 randomized controlled trials and observational studies, indicated that surgical masks and N95 respirators were supportive measures offering the most consistent protection.^9^ However, these reviewers also concluded that the assessment of such measures was difficult.^9^ With the COVID-19 pandemic, it has become necessary to widely implement supportive protection using masks or N95 respirators, which may subsequently enable further multiregional or multinational studies on these issues. Notably, Holshue et al^10^ reported the first case of COVID-19 in the United States from a stool specimen that tested positive using a real-time reverse-transcriptase polymerase chain reaction (rRT-PCR) assay.

Because the full picture of the biological features of SARS-CoV-2 has yet to be elucidated, it is prudent to consider more routes of potential risk. In summary, the protective role of both N95 and medical masks in other diseases could be translated to the fight against SARS-CoV-2, and their specific contribution remains to be quantified.
